# Cooperative effects in DNA-functionalized polymeric nanoparticles

**DOI:** 10.1039/d5nr01614b

**Published:** 2025-08-13

**Authors:** Paraskevi Gaki, Andrey S. Klymchenko

**Affiliations:** a Laboratoire de Bioimagerie et Pathologies, UMR 7021 CNRS, Faculté de Pharmacie, Université de Strasbourg 67401 Illkirch France andrey.klymchenko@unistra.fr; b BrightSens Diagnostics SAS 11 Rue de l'Académie 67000 Strasbourg France

## Abstract

DNA-functionalized nanoparticles (NPs), called spherical nucleic acids (SNAs), have attracted considerable attention due to their unique properties and numerous applications. In particular, DNA-functionalized dye-loaded polymeric NPs (DNA-NPs), owing to their exceptional fluorescence brightness, have emerged as powerful nanomaterials for the ultrasensitive detection and imaging of nucleic acids. Herein, we addressed a fundamental question unexplored for polymeric DNA-NPs: how does the dense packing of oligonucleotides on the particle surface impact their capacity to specifically hybridize with complementary sequences? Using Förster resonance energy transfer (FRET) between DNA-NPs and labelled complementary strands, we found that the DNA on the surface of the NPs exhibits dramatic enhancement in duplex stability compared to free DNA duplexes (>20 °C). This effect increases at higher densities of coding DNA on the NP surface, which suggests that DNA cooperativity is responsible for the enhancement in duplex stability. For example, 8 nt DNA duplexes were perfectly stable at RT on the surface of DNA-NPs. Furthermore, these DNA-NPs preserve the capacity to distinguish mutations, even at the single-nucleotide level within a 21 nt sequence, when an appropriate hybridization temperature is used. The hybridization between DNA-NPs and the complementary sequences proceeds on the min time scale at probe and target concentrations of ≥10 and ≥100 pM, respectively. Below these, this diffusion-controlled process becomes too slow, indicating the fundamental limitation in DNA/RNA sensing assays that require sufficiently high nanoprobe concentration. The present study sheds light on the capacity of DNA-NPs to specifically hybridize with the target sequences and provides insights into the development of nucleic acid sensing assays.

## Abstract 2

This work addresses the fundamental question of how the dense packing of oligonucleotides on the surface of polymeric nanoparticles impacts their capacity to specifically hybridize with complementary sequences.

## Graphical abstract

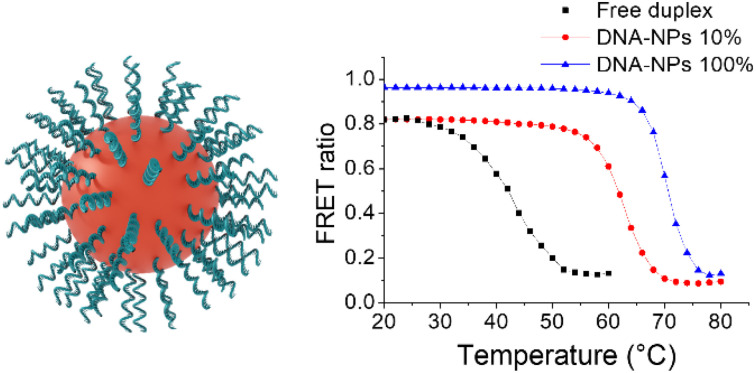

## Introduction

Luminescent nanoparticles (NPs) gained important interest due to their multiple unique characteristics, such as a high surface to volume ratio, surface multi-functionality, and capacity to encapsulate large quantities of cargo.^1,2^ Their exceptional brightness provides a high signal to noise ratio in biosensing and bioimaging, which is important for diagnostic applications.^3,4^ Luminescent NPs can be categorized into inorganic, such as quantum dots (QDs),^5^ dye-loaded silica NPs,^6^ metal nanoclusters,^7^ metal–organic framework NPs,^8^ and carbon dots,^9^ and organic, such as conjugated polymer NPs,^10–12^ aggregation-induced emission (AIE) NPs,^13–15^ dye-loaded polymers^16,17^ and lipid^18^ NPs. Among them, dye-loaded polymeric NPs are of particular interest because they provide high stability in biological media, good capacity to encapsulate functional cargoes, rich surface chemistry and biocompatibility.^16,19,20^ Early reports showed that polymeric NPs could encapsulate conventional dyes only at ∼1 wt%, whereas higher loadings lead to aggregation-caused quenching and, thus, loss in particle brightness.^19,21,22^ We addressed this problem by proposing bulky hydrophobic counterions as insulators, preventing dye aggregation and ensuring efficient loading without dye leakage in biological media.^23–25^ This approach ensured efficient dye emission even at dye loadings reaching 33 wt% of particle mass,^26^ which resulted in exceptional particle brightness^4^ and unique light-harvesting properties.^27^

A particular class of NPs is those functionalized with nucleic acids,^28^ which Mirkin and co-workers named spherical nucleic acids (SNAs).^29,30^ They are spherical nanoparticles coated with nucleic acids at high density, which endows them with unique properties, such as programmable assembly, high stability against enzymes and high colloidal stability.^30,31^ These properties enabled the preparation of new types of self-assembled materials as well as led to new technologies for nucleic acid biosensing, bioimaging and diagnositcs,^3,31–34^ gene regulation,^35–38^ drug delivery,^39,40^ precision medicine^41,42^ and immunotherapy.^39,43^ A large variety of DNA-functionalized NPs was synthesized based on gold^29,30,33,35,44,45^ and other noble metals,^46–48^ metal oxide (magnetic) NPs,^28,35,49–51^ liposomes,^39,52^ micelles,^53^ polymeric NPs,^32,39,40,43,54,55^*etc*. In the field of polymeric DNA-functionalized NPs, a lot of efforts were focused on the preparation of DNA-polymer conjugates,^56^ with the further formulation of amphiphilic block copolymer SNA,^40,57^ although the modification of hydrophobic polymers in the form of NPs with DNA remains challenging. We reported the preparation of DNA-functionalized polymeric NPs, which included a special polymer design that ensures the high exposure of azide groups on the surface of the NPs.^55^ The latter enabled the effective grafting of short DNA at high density, which yielded polymer-based SNA. Due to their high brightness and light-harvesting properties, they enabled the amplified FRET-based detection of DNA fragments^55^ and microRNA,^58^ with sensitivity down to single-molecule hybridization,^26^ using a robust ratiometric output, compatible with smartphone detection.^59^ More recently, these NPs enabled amplified fluorescence *in situ* hybridization for RNA imaging in cells,^60^ as well as the zeptomole-sensitive detection of viral RNA in combination with magnetic beads.^61^

The intriguing question in the field of SNA is their cooperativity and multivalence due to the presence of numerous repeats of DNA with the same orientation and high local concentration. Multivalency in NPs is a generic phenomenon,^62^ which is known to increase their affinity to the corresponding targets by the multiple presentation of small molecular ligands,^63–65^ protein ligands,^66^ and antibodies,^67^ and it was extensively supported by theoretical modeling.^68,69^ Regarding SNA, the early studies on SNA based on gold NPs suggested that a higher density of oligonucleotides on the surface of SNA led to higher duplex stability (higher melting point, *T*_m_) and sharper melting transition curves due to cooperative effects.^70^ Overall, the gain in the *T*_m_ values was ∼5 °C for gold SNA *vs.* free DNA duplex.^29^ Further studies on cross-linked micellar SNA reported an increase in *T*_m_ by 16.5 °C for DNA duplexes between two SNA *vs.* free DNA duplex,^71^ although the hybridization of multiple duplexes between the two NPs probably contributed to this significant increase in *T*_m_. A recent study showed that the density of single-stranded DNA on the surface of magnetic nanoparticles plays a crucial role in their organization and cooperative dynamics.^49^ At higher densities, DNA strands adopted a brushed organization with cooperative dynamics, favoring stronger hybridization, which is important for magnetic biosensing.^49^ To the best of our knowledge, the cooperative effect has not been well studied for DNA-functionalized polymeric NPs. In this respect, we were particularly interested to explore the extent of the DNA cooperativity effect in dye-loaded polymeric NPs, the impact of the surface density of the coding sequence, specificity of the hybridization and the sensitivity to mutations. All these questions are essential for the further utilization of polymeric SNA in nucleic acid detection and imaging applications, where these NPs have already shown significant promise.^26,55,61^

In the present work, we addressed a fundamental unexplored question on how a high density of oligonucleotides grafted on the surface of polymeric DNA-NPs impacts their capacity to specifically hybridize with complementary sequences and the stability of the obtained duplexes. Using a FRET approach, we found a remarkable enhancement in the stability of DNA duplexes on the surface of polymeric NPs caused by the DNA cooperativity effect. DNA-NPs can distinguish well a single mutation in their complementary sequences and hybridize at the min time scale when the nanoprobe/target concentrations are at the high pM level (10–100 pM). These results show the strong potential of DNA-NPs as DNA/RNA nanoprobes and provide insights into the development of next-generation detection assays.

## Materials and methods

### Materials

Rhodamine B octadecyl ester tetrakis(pentafluorophenyl)borate (R18/F5-TPB) was synthesized by ion exchange and purified by column chromatography, as explained previously.^24^ PEMA-AspN3-5% polymer was synthesized as described previously.^26^ Sodium phosphate dibasic dihydrate (>99.0%) and sodium phosphate monobasic (>99.0%) were both purchased from Sigma-Aldrich and used to prepare 20 mM phosphate buffer at pH 7.4. Millex®-GP Filters (pore size of 0.22 μm and diam. of 33 mm) and Amicon Centrifugal filters (0.5 mL, 100 kDa) were purchased from Sigma-Aldrich. Ultrapure DNase/RNase-Free water (Invitrogen) was used in all experiments. Dulbecco's Phosphate Buffered Saline (PBS) and low binding microcentrifuge tubes were purchased from Dutscher.

### Nucleic acids sequences

Lyophilized single strand DNA and RNA sequences were purchased from Biomers GmbH and Eurogentec, dissolved in ultrapure DNase/RNase-Free water, aliquoted, and stored at −20 °C for further experiments. The oligonucleotide sequences used in this study are shown below.

Sur NPs: 5′ CCC-AGC-CTT-CCA-GCT-CCT-TGA – (DBCO) 3′

A20-DBCO: 5′ AAA-AAA-AAA-AAA-AAA-AAA-AA – (DBCO) 3′

8-Nt acceptor: 5′ (Atto 665) – TCA-AGG-AG 3′

12-Nt acceptor: 5′ (Atto 665) – TCA-AGG-AGC-TGG 3′

21-Nt acceptor: 5′ (Atto 665) – TCA-AGG-AGC-TGG-AAG-GCT-GGG 3′

21-Nt acceptor_1MUT: 5′ (Atto 665) – TCA-AGG-AGC-TCG-AAG-GCT-GGG 3′

21-Nt acceptor_3MUT: 5′ (Atto 665) – TCA-AAG-AGC-TCG-AAG-GGT-GGG 3′

Donor-21: 5′ CCC-AGC-CTT-CCA-GCT-CCT-TGA – (Cy3) 3′.

### Synthesis of fluorescent nanoparticles

50 μL of polymer (PEMA-AspN3-5%) solution in acetonitrile (2 mg mL^−1^ containing R18/F5-TPB at 50 wt% relative to the polymer, meaning 33 wt% to particle mass) was added quickly using a micropipette to 450 μL of 20 mM phosphate buffer (PB), pH 7.4 at 21 °C under shaking (Thermomixer Comfort, Eppendorf, 1150 rpm). Then, the residues of acetonitrile were evaporated under reduced pressure.

### Synthesis of DNA-functionalized nanoparticles (DNA-NPs)

Aliquots of DBCO-sequence (20 μM in the final reaction mixture) were added to 100 μL of the corresponding nanoparticles. In the case of NPs with different percentages of coding sequence, the total concentration was 20 μM. Then, they were mixed carefully, microcentrifuged and kept overnight (21 h) at 40 °C in an incubator chamber without shaking and protected from light. Afterwards, the mixtures were cooled to room temperature. These reaction mixtures were purified by centrifugation using centrifuge filters (Amicon, 0.5 mL, 100 kDa) on 1000 rcf at 20 °C for 5 min. The centrifugation procedure was repeated 4 times to remove the non-reacted oligonucleotides. The obtained DNA-functionalized nanoprobes were diluted to 1 nM concentration and kept in the dark at 4 °C.

### Characterization of nanoparticles

Dynamic light scattering (DLS) measurements were performed on a Zetasizer Nano ZSP (Malvern Instruments S.A.). The Zetasizer software, providing standard cumulates and size distribution by volume analysis, was used to characterize nanoparticles by DLS. For the data analysis, the following parameters were used: for the solvent (water) – temperature of 25 °C, refractive index (RI) of 1.33, and viscosity of 0.8872 cP. The nanoparticles were assumed to be all homogenous and spherical in shape. Absorption spectra were recorded on a Cary 5000 Scan UV–vis spectrophotometer (Varian). Emission spectra were recorded on an FS5 Spectrofluorometer (Edinburg Instruments) and FluoroMax-4 Spectrofluorometer (HORIBA Scientific) equipped with a 350B Thermoelectric Temperature Controller (Newport) for measurements requiring strict temperature control. For the standard recording of fluorescence spectra, the excitation wavelength was set at 530 nm. The fluorescence spectra were corrected for detector response and lamp fluctuations.

### DNA cooperativity study on the surface of DNA-NPs

For FRET between DNA-modified NPs and different concentrations of acceptor-bearing sequence of 21 nucleotides, aliquots of 21-nt acceptor of varying concentrations were added to 100% coding (100% survivin capture sequence) or 10% coding (10% survivin capture sequence & 90% A20 sequence) DNA-NPs (50 wt% of R18/F5-TPB dye with respect to the polymer, meaning 33 wt% of the total mass of the NPs) in Mg buffer, to a final concentration of 100 pM of DNA-NPs in the final volume of 300 μL in low binding microcentrifuge tubes. The Mg buffer contained 20 mM of phosphate buffer along with 12 mM MgCl_2_ and 30 mM NaCl at pH 7.4. The mixtures were incubated at 40 °C for 20 min and protected from light. Afterwards, they were cooled to room temperature. Finally, steady-state spectra were recorded.

### Thermal stability of duplexes of DNA-NPs with acceptor-sequences

In the thermal stability studies using FRET between the DNA-modified NPs and acceptor-bearing sequence, aliquots of 8-nt acceptor or 12-nt acceptor at a 10 nM final concentration were added to 100% coding (100% survivin capture sequence) or 10% coding (10% survivin capture sequence & 90% A20 sequence) DNA-NPs (50 wt% of R18/F5-TPB dye with respect to the polymer, meaning 33 wt% of the total mass of the NPs) in Mg buffer to a final concentration of 100 pM of DNA-NPs in a final volume of 600 μL in low binding microcentrifuge tubes. The mixtures were incubated at 40 °C for 20 min and protected from light. Afterwards, they were cooled to room temperature. For the mixtures, their fluorescence spectra were recorded at the starting point of 20 °C at 2 °C intervals up to 60 °C (for the samples with 8-nt acceptor) or 80 °C (for the samples with 12-nt acceptor). The temperature was increased gradually using a Peltier-based temperature control connected to the fluorometer.

### Thermal stability of individual DNA duplexes

In the thermal stability studies using FRET between a single dye donor attached to the same DNA sequence that is present on the NPs (donor-21) and acceptor-bearing sequence, aliquots of 8-nt acceptor or 12-nt acceptor at a 10 nM final concentration were added to donor-21 at a 3.3 nM final concentration in Mg buffer to a final volume of 600 μL in low binding microcentrifuge tubes. The mixture containing 8-nt acceptor was incubated at 4 °C for 1 h, whereas the mixture containing 12-nt acceptor was incubated at 40 °C for 20 min and both protected from light. The latter was cooled to room temperature before starting the measurements. For the mixture with 8-nt acceptor, a temperature range of 6 °C to 40 °C was selected, and for the mixture with 12-nt acceptor, a temperature range of 20 °C to 60 °C was selected. Fluorescence spectra were recorded at the lowest temperature at 2 °C intervals until the highest temperature. The temperature was increased gradually using a Peltier-based temperature control connected to the fluorometer. The melting temperature for 8mer and 12mer was estimated online using the “OligoAnalyzer” tool of IDT (https://www.idtdna.com/calc/analyzer), which took into account the DNA concentration (10 nM) and composition of the Mg buffer.

### Sensitivity to mutations of DNA-NPs

To study the sensitivity of the NPs to mutations, FRET between the DNA-modified NPs and acceptor-bearing sequence with zero, one and three point mutations was used. Aliquots of 21-nt acceptor, 21-nt acceptor_MUT1 and 21-nt acceptor_MUT3 at a 10 nM final concentration were added to 100% coding (100% survivin capture sequence) or 10% coding (10% survivin capture sequence & 90% A20 sequence) DNA-NPs (50 wt% of R18/F5-TPB dye with respect to the polymer, meaning 33 wt% of the total mass of the NPs) in Mg buffer to a final concentration of 100 pM of DNA-NPs in a final volume of 600 μL in low binding microcentrifuge tubes. The mixtures were incubated at 40 °C for 20 min and protected from light. Afterwards, they were cooled to room temperature. For the mixtures, their fluorescence spectra were recorded at the starting point of 20 °C at 2 °C intervals up to 70 °C. The temperature was increased gradually using a Peltier-based temperature control connected to the fluorometer.

### Kinetics of hybridization of DNA-NPs with acceptor-sequences

Aliquots of 21-nt acceptor were added to 100% coding (100% survivin capture sequence) DNA-NPs (50 wt% of R18/F5-TPB dye with respect to the polymer, meaning 33 wt% of the total mass of the NPs) in Mg buffer, to a final volume of 600 μL in the plastic cuvettes that were used to record the spectra, as to not waste time. The components were used in a 1 NP : 10 acceptor ratio, at different concentrations, starting from 1 pM DNA-NPs mixed with 10 pM 21-nt acceptor. Fluorescence spectra were recorded for 10 min at the rate of one spectrum per minute. Before proceeding to the analysis, all spectra were smoothed using Adjacent-Averaging with 5 Points of Window.

### FRET ratio calculation

The FRET ratio was calculated using the following equation:FRET ratio = *I*_A_/(*I*_A_ + *I*_D_),where *I*_A_ is the fluorescence intensity of the acceptor and *I*_D_ is the fluorescence intensity of the donor (DNA-NPs or single dye donor).

## Results and discussion

### DNA-NPs and their hybridization studied by FRET

**Fig. 1:**
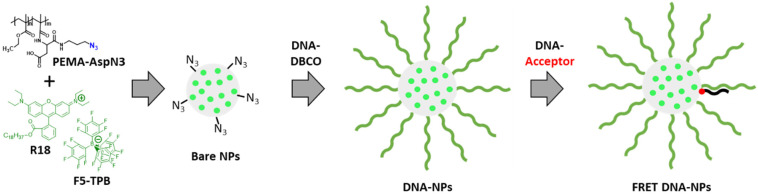
Chemical structures of the PEMA-AspN3 polymer and dye R18/F5-TPB, their nanoprecipitation into bare dye-loaded polymeric NPs with functional azide groups, functionalization of the latter into DNA-NPs and hybridization of the complementary stands with a FRET acceptor.

The designed DNA-NPs are comprised of a dye-loaded polymeric core and a single-stranded DNA shell. The core is based on poly(ethyl methacrylate)-*co*-methacrylic acid bearing aspartic acid with azide (PEMA-AspN3, 5% methacrylic acid)^26^ and loaded at 33 wt% (to particle mass) with an ion pair of hydrophobic rhodamine (R18) with the bulky hydrophobic counterion tetrakis(pentafluorophenyl)borate (F5-TPB) to avoid aggregation-caused quenching (Fig. 1). The dye molecules of the core act as the energy donor in the Förster resonance energy transfer (FRET) pair and can transmit energy to an acceptor dye on the surface.^26,55^ Given that the fluorescence lifetime of the R18/F5-TPB dye inside this type of NPs is ∼2 ns,^27^ the ultrafast excitation energy migration within the donor dyes on the 30 fs time scale allows efficient energy transfer from the NP core to the surface where the acceptors are located. To obtain DNA-NPs, the NP core bearing azide groups at the surface is functionalized with a single-stranded capture oligonucleotide bearing DBCO *via* a strain-promoted cycloaddition reaction (Fig. 1).^26,55^ A DNA fragment mimicking the corresponding mRNA segment of survivin, which is an inhibitor of the apoptosis protein widely expressed in malignant cells and an important cancer marker, was selected as the target complementary to the DNA capture sequence of DNA-NPs. To study how the composition of the DNA shell of the NPs affects the hybridization with the complementary target sequence, we exploited FRET from the NP core to the acceptor-modified target hybridized on the DNA-NP surface to the capture oligonucleotide (Fig. 1). As the FRET acceptor, we used ATTO-665, which exhibits a good match with the R18/F5-TPB dye as the FRET donor, with a Förster radius of 6.7 nm.^26^

**Fig. 2:**
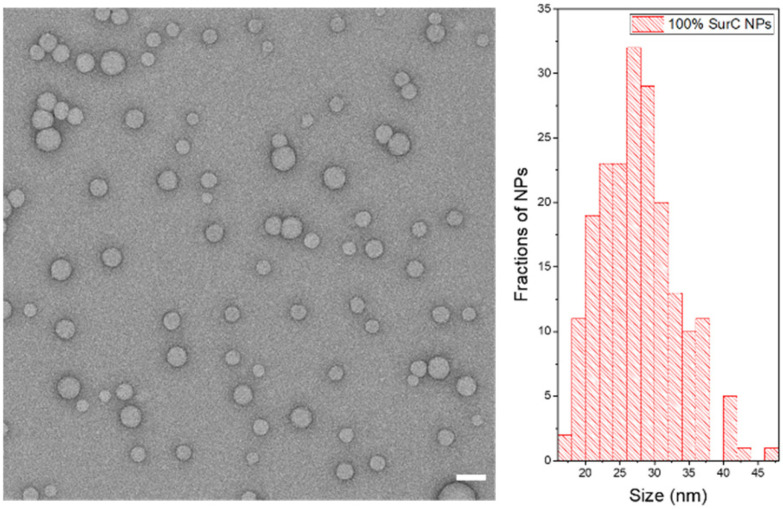
TEM image (left) and size distribution diagram (right) of PEMA-AspN3 NPs functionalized at 100% with a survivin capture coding sequence and loaded with 33 wt% of R18/F5-TPB dye (with respect to total NP mass). Scale bar: 50 μm.

The core of the nanoparticle was prepared by nanoprecipitation of PEMA-AspN3 and R18/F5-TPB to yield NPs with a size of 33 nm and low polydispersity according to DLS (Table S1). TEM confirmed the spherical shape and small uniform size (23 ± 4 nm) of the obtained NPs (Fig. S1). They showed a single absorption and the typical emission spectrum for R18 dye (Fig. S2). Then, the obtained NPs were functionalized with different fractions of coding sequence. As the coding sequence on the DNA-NPs, single-stranded DNA complementary to a part of survivin mRNA, an important cancer marker, was selected. As a non-coding oligonucleotide, single-stranded A20 was used. The DNA-NPs were prepared with 100% and 10% of coding sequence and 0% and 90% of A20 sequence, respectively. After functionalization with oligonucleotides, their hydrodynamic diameter increased by 12–13 nm (Table S1), which corresponded to the presence of an additional DNA shell. TEM showed a similar spherical shape (Fig. 2 and Fig. S3) and slightly increased diameter of 28 ± 5 and 32 ± 6 and for 100% and 10% coding DNA-NPs, respectively (Table S1). Their absorption and emission spectra remained unchanged after functionalization (Fig. S4 and S5). The fluorescence quantum yield of the obtained DNA-NPs (10% coding) was 53%, which was consistent with earlier works on PEMA-AspN3 NPs.^26^

**Fig. 3:**
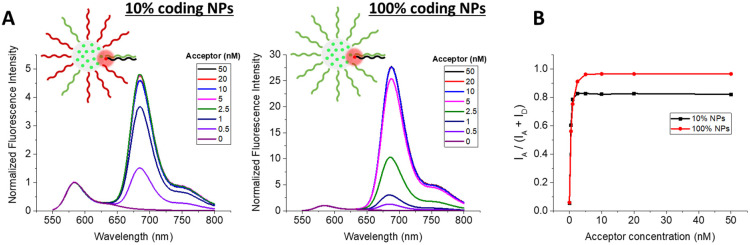
DNA hybridization in DNA-NPs with varying surface density of coding sequences studied by FRET. (A) Fluorescence spectra of 100% coding (100% survivin capture sequence) and 10% coding (10% survivin capture sequence, 90% A20 sequence) DNA-NPs (50 wt% of R18/F5-TPB dye with respect to the polymer, meaning 33 wt% of the total mass of the NPs) hybridized at 40 °C for 20 min, with varying concentrations of acceptor dye bearing a DNA sequence of 21 nucleotides, representing part of the survivin target, thus being fully complementary to the capture sequence on the DNA-NPs. For this experiment, 100 pM of DNA-NPs were hybridized with 0.5–50 nM of the acceptor-sequence. Negative control: DNA-NPs without the addition of the acceptor-sequence. Mg buffer was systematically used. Spectra were measured at 530 nm excitation at RT. (B) FRET ratio for the 100% coding and 10% coding DNA-NPs, measured by varying the concentration of the acceptor-sequence.

An increasing concentration of target (complementary) DNA sequence labeled with acceptor dye (Atto-665) was added to each type of DNA-NPs and their fluorescence spectra were recorded (Fig. 3A). In all the studied DNA-NPs, an increase in the DNA-acceptor concentration led to a higher signal of the long-wavelength band corresponding to the FRET acceptor. Using the fluorescence spectra, the FRET ratios were calculated for all conditions using the formula *I*_A_/(*I*_A_ + *I*_D_), where *I*_A_ and *I*_D_ are the peak intensities of the acceptor and donor, respectively, in the fluorescence spectrum. The FRET ratio gives semi-quantitative information on the FRET efficiency, and consequently the efficacy of hybridization between the capture DNA on the NPs and the target. In both studied DNA-NPs, a steep increase in the FRET ratio was observed with an increase in the DNA-acceptor concentration (Fig. 3B). At higher DNA-acceptor concentrations, the curves reached a plateau, but the saturation values of the FRET ratio were higher for the DNA-NPs with a higher fraction of coding sequence. In the case of the 10% and 100% coding DNA-NPs, the observed saturations occurred at 2 and 5 nM DNA-acceptor, respectively. This saturation suggested that the DNA-NPs reached their maximum capacity to hybridize the DNA-acceptor. However, the saturating concentration only increased by 2.5-fold for a 10-fold increase in the fraction of coding sequence, which indicates that in the case of 100% coding DNA-NPs, the hybridization is not complete. Assuming that in the case of 10% coding DNA-NPs the DNA hybridization is complete, we could calculate the number of DNA grafted per NP. Considering that the DNA-NP concentration was 100 pM, the number of hybridized DNA-acceptors was 20. Given that this corresponds to 10% of all the DNA strands, the total number of DNA strands per NP is expected to be 200. This value is close to that estimated for similar dye-loaded polymeric NPs.^26,55^ Even though for 100% coding DNA-NPs the saturation was reached at higher concentrations, it was still below the expected value (25% of the expected value), suggesting that only 25% of the capture sequences were hybridized in this case, whereas further hybridization was probably blocked because of the steric hindrance produced by the increased density due to the newly formed DNA duplexes. Indeed, considering 200 DNA strands per 23 nm NPs, the area per DNA is ∼8 nm^2^. This ensures a relatively short DNA–DNA spacing of ∼3 nm, which produces a brushed configuration with strong repulsion between DNA chains, leading to steric effects. The above-mentioned results show that the hybridization capacity depends on the density of the coding nucleic acids, where a larger fraction of coding sequence helps to hybridize a larger amount of the targets, but the hybridization is probably limited by the steric hindrance at the surface due to the high nucleic acid density.

To verify that our DNA-NPs are capable of hybridizing with their target even in complex biological media, we studied the effect of fetal bovine serum (FBS), which is a complex mixture of proteins from blood. We performed the hybridization of DNA-NPs with 100% survivin-encoding in the same conditions as above (20 min at 40 °C) with 10 nM 21-nt survivin fragment in our buffer without (control) and with 10% FBS. A strong FRET signal was observed in the case of the FBS-containing medium with FRET ratio values close to that of the control (Fig. S6), indicating that our DNA-NPs can operate in a complex biological medium originating from blood. This result is in line with our earlier work on FRET-based nanoprobes, which were also compatible with FBS.^55^

### Thermal stability of the duplexes in DNA-NPs

The high density of DNA on the NP surface is expected to lead to cooperative phenomena, which could increase the stability of hybridization compared to that of the free single-stranded sequences. For this reason, we studied the thermal stability of the duplexes on the surface of the DNA-NPs in comparison to the free DNA duplexes. Two FRET systems were used, as follows: (i) DNA-NPs acting as the energy donor and dye-bearing target sequence as the acceptor and (ii) the DNA capture sequence, similar to the DNA-NPs, bearing donor dye and the same target DNA-acceptor. Thus, the DNA sequences used are the same in these two cases and the differences in the hybridization can be directly attributed to the effect of the confinement of DNA on the NP surface.

DNA-NPs were prepared with 100% and 10% coding sequences on their surface, as mentioned above. They were hybridized with acceptor dye-labeled target sequences of different lengths of 8 and 12 nt. These short target sequences are complementary to the end attached to the NP, where the acceptor dye is placed next to the donor dye-loaded core of DNA-NPs. In the control without DNA-NPs, the single-stranded DNA bearing the donor dye (Atto 665) was hybridized with the acceptor dye-bearing target sequence of 8 and 12 nt. In both the DNA-NP and single-stranded DNA donor systems, we used an excess of acceptor-sequence at a constant concentration of 10 nM. The above-mentioned hybridization was performed by an annealing procedure, where the sample was subjected to a heating step, which disrupts potential secondary structures by breaking all hydrogen bonds, followed by a cooling step, which facilitates the formation of new hydrogen bonds between complementary oligonucleotides.

To evaluate the thermal stability of the duplexes, we started by calculating the melting temperature of the free DNA duplexes (*T*_m_). In the case of 8mer and 12mer at a 10 nM concentration in Mg buffer, their calculated *T*_m_ was 15 °C and 43 °C, respectively. It is important to keep in mind that these are approximations and that their real melting temperature depends on many factors, including the buffer composition.

**Fig. 4:**
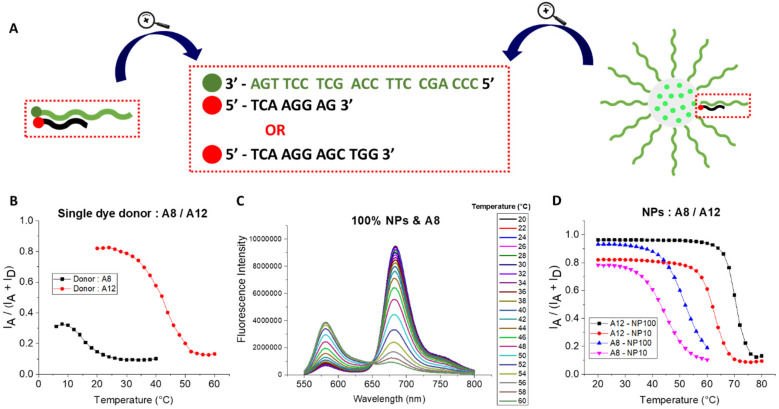
Thermal stability of the duplexes. (A) Schematic representation of the single-stranded donor and acceptor-sequence pairs and the NP and acceptor-sequence pairs. The oligonucleotide sequences attached to the donor and acceptor are presented. (B) FRET ratios for increasing temperature for the pairs of a single dye donor: 8-nt acceptor (A8, in black) and 12-nt acceptor (A12, in red). In this experiment, 3.3 nM of the donor-sequence was hybridized with 10 nM of the acceptor-sequence. (C) Fluorescence spectra of 100% coding DNA-NPs hybridized with the 8-nt acceptor and subjected to increasing temperatures from 20 °C to 60 °C. (D) FRET ratios for 10% and 100% coding DNA-NPs, hybridized with the 8-nt and 12-nt acceptors. In this experiment, 100 pM of DNA-NPs was hybridized with 10 nM of the acceptor-sequence. The temperature was increased from 20 °C to 60 °C (for 10% coding DNA-NPs) or to 80 °C (for 100% coding DNA-NPs). Mg buffer was systematically used. Spectra were measured at 530 nm excitation.

For each duplex sample, its fluorescence spectrum was recorded at the starting point of 20 °C at 2 °C intervals up to 60–80 °C (Fig. 4 and Fig. S7–S11). The temperature was increased gradually using a Peltier-based temperature control, connected to the fluorometer. The resulting fluorescence spectra were used to calculate the FRET ratios. Subsequently, the FRET ratios were plotted against the temperature to obtain the melting curves. At the starting point for all the samples, a high value of FRET ratio was observed, indicating that duplexes were formed. In the case of the free DNA duplex with 8-nt acceptor, the starting temperature was set at 6 °C, given that its melting temperature was low and we intended to see the transition. The melting curves of the free DNA duplexes with 8- and 12-nt acceptors showed the classical melting profiles (Fig. 4B). At lower temperatures, the FRET ratio remained high because the sequences were in double-stranded form. When reaching close to their expected melting temperature, a decrease in the FRET ratio was observed, signifying the dissociation of the duplex.

In the case of the FRET systems with the DNA-NPs, their fluorescence spectra showed strong temperature dependence, where the initially high signal of the FRET acceptor decreased at higher temperatures, whereas the donor emission increased, indicating the loss of FRET due to disruption of the DNA duplexes (Fig. 4C). In the obtained melting curves for 10% and 100% coding DNA-NPs with 8- and 12-nt acceptor, high FRET ratio values were observed at lower temperatures (Fig. 4D), suggesting the presence of DNA duplexes. An increase in temperature resulted in a sigmoid response, with the sharp decrease in the FRET ratio corresponding to the DNA duplex melting. The melting temperature was characteristic for each sample, which increased in the following order: 10% DNA-NPs with 8-nt acceptor <100% DNA-NPs with 8-nt acceptor <10% DNA-NPs with 12-nt acceptor <100% DNA-NPs with 12-nt acceptor. Thus, as expected, the longer sequences melted at a higher temperature. More importantly, an increase in the density of coding sequences on the DNA-NP surface increased the melting point (Fig. 4D). Another important finding is that melting curves obtained for DNA-NPs drastically shifted towards higher temperatures and appeared sharper compared to that of the free DNA duplexes (Fig. 4D). In the case of the 8-nt acceptor, 10% and 100% DNA-NPs showed a shift of 18 °C and 26 °C, respectively, in their melting curve to higher values compared to the corresponding free duplex. In the case of the 12-nt acceptor, the melting curve shifted by ∼20 °C and ∼ 28 °C for 10% and 100% coding DNA-NPs, respectively. Moreover, in the case of both 8- and 12-nt acceptors, the melting curve was sharper for 100% *vs.* 10% coding DNA-NPs. All these results show the strong DNA cooperativity effect, where the close proximity of the coding DNA on the NP surface boosts the stability of the duplex and sharpness of the melting curve. Indeed, the previous works by Mirkin and co-workers showed that the DNA cooperativity in SNA based on gold NPs made their melting curves sharper compared to individual DNA strands.^29,70^ The enhancement in the obtained *T*_m_ value is significantly higher than that reported for the classical example of gold SNA, where only an enhancement of ∼5 °C was reported for SNA *vs.* free DNA duplex.^70^ This effect is even stronger compared to SNA (to cross-liked micelles) connected by DNA duplexes (16.5 °C).^71^ Moreover, the observed enhancement in the duplex stability with an increase in the density of the coding sequence (by 8 °C from 10% to 100% of coding sequence density) is in line with the earlier report on gold SNA, where *T*_m_ increased by 3.1 °C with an increase in coding DNA density from 33% to 100%.^70^ Thus, SNA based on polymeric NPs exhibit a remarkably high DNA cooperativity effect, surpassing classical examples of gold SNA, which enables the formation of stable duplexes, even for relatively short DNA sequences down to 8 nt, which are now stable at RT.

We further explored the importance of the distance between the coding sequence and the particle surface. PolyA spacers of different lengths (0, 5, 10 and 40 nt) were placed between the survivin capture sequence and the points of grafting to the NP surface. 100% coding DNA-NPs were prepared and studied with 12-nt acceptor at varying temperatures. We found that an increase in the spacer length caused a sequential decrease in the *T*_m_ values (Fig. S12). The lowest *T*_m_ value was observed for the 40 nt polyA spacer, corresponding to an ∼20 °C decrease in *T*_m_ compared to DNA-NPs without the polyA spacer. Nevertheless, the *T*_m_ values for the 40 nt polyA spacer were still slightly higher compared to that for the free 12-nt DNA duplex. With an increase in the distance of the coding sequence from the NP surface, the distance between the grafted DNA stands should increase. The latter should result in the effect of dilution of the capture sequences, which is expected to decrease the cooperative effects, thus leading to a decrease in *T*_m_. Secondly, placing the coding sequence away from the surface also decreases the alignment of the DNA sequences because of the higher steric freedom, which can further decrease the cooperativity effects (*i.e. T*_m_), given that the hybridization sites are less aligned in space. These conclusions are in line with a recent work, showing that decreasing the density of DNA grafted to the surface of magnetic NPs leads to a change from brushed to coiled configuration, thus decreasing the cooperative effects.^49^ One should also note that the FRET efficiency at low temperatures dropped significantly for the 40 nt spacer, which was expected given that its length (13.6 nm in the fully stretched conformation) is larger than the Förster radius (6.7 nm). In reality, 40 nt polyA is probably not fully stretched in the brushed configuration, which explains the still detectable FRET, allowing us to measure the *T*_m_ of the DNA-NP system.

**Fig. 5:**
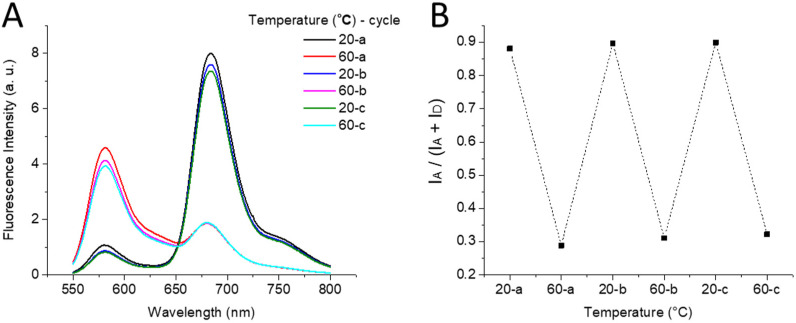
Reversibility of FRET in heating–cooling cycles for DNA-NPs (100 pM), 100% coding with the survivin capture sequence in the presence of the 8-nt acceptor (10 nM): emission spectra (A) and corresponding FRET ratios (B). Spectra were measured at 530 nm excitation.

Next, it was important to understand whether our DNA-NP FRET system with thermally controlled hybridization is fully reversible, thus allowing full recovery of the DNA-NPs. For this purpose, we performed 3 sequential heating–cooling cycles and recorded the fluorescence spectra of 100% coding DNA-NPs with the 8-nt oligonucleotide acceptor. The initial step of heating led to the expected loss of the FRET signal, while further cooling resulted in complete recovery of FRET with a nearly superimposable emission spectrum (Fig. 5). Further heating–cooling cycles produced identical switching of FRET, suggesting that our DNA-NP system is fully reversible and recoverable.

### Sensitivity to mutations

To be useful for RNA/DNA sensing in molecular diagnostics, our DNA-NPs should present a high level of sequence specificity, ideally with the capacity to detect a single mutation in the target sequence. To understand the effect of mutations, the DNA-NPs were investigated in the same FRET-based format. We designed sequences with 1 and 3 point mutations based on an acceptor dye-labeled target sequence of 21 nt, fully complementary to the capture sequence on the DNA-NPs. The single mutation was introduced in the middle of the sequence (A21_1MUT), whereas the 3 mutations (A21_3MUT) were spread across the sequence, keeping approximately the same length in the formed short complementary fragments. This should in theory split the long sequence in shorter ones and decrease the melting temperature.

**Fig. 6:**
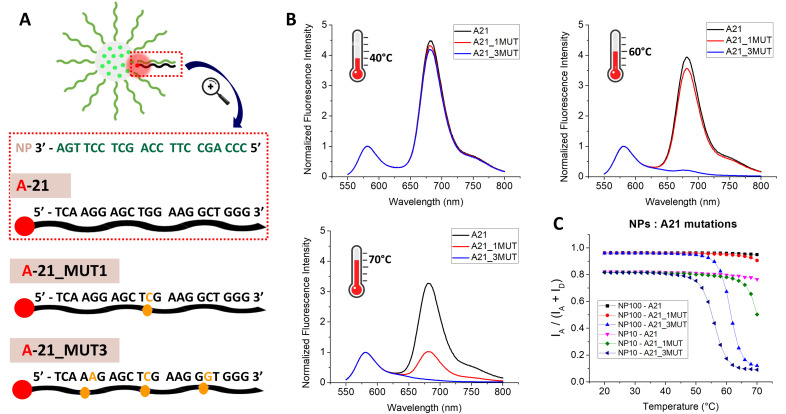
Sensitivity to mutations. (A) Schematic representation of the NP hybridized with acceptor-sequences each containing zero (A-21), one (A-21_MUT1) or three (A-21_MUT3) point mutations, as indicated by the yellow nucleotides. (B) Normalized fluorescence spectra for 10% coding DNA-NPs hybridized with acceptor sequences with zero (in black), one (in red) or three (in blue) point mutations at three different temperatures of 40 °C, 60 °C and 70 °C. (C) FRET ratios *vs*. tempetature for 10% and 100% coding DNA-NPs, hybridized with acceptor-sequences with zero, one or three point mutations. In these experiments, 100 pM of DNA-NPs was hybridized with 10 nM of the acceptor-sequence. The temperature was increased from 20 °C to 70 °C. Mg buffer was systematically used. Spectra were measured at 530 nm excitation.

DNA-NPs with 100% and 10% coding sequences were hybridized with three types of acceptor sequences with a different number of mutations. The DNA-NPs were annealed with their complementary sequences labelled with the FRET acceptor, as explained above to achieve complete hybridization. For all the studied pairs of DNA-acceptor with DNA-NPs, their fluorescence spectrum was recorded at the starting point of 20 °C at 2 °C intervals up to 70 °C (Fig. S13–S18). At 20 °C, the DNA-NPs with the same density of coding sequences presented almost the same high FRET ratio values, indicating the presence of stable duplexes (Fig. 6). In the case of the 10% coding DNA-NPs, the duplex with A21_3MUT started to melt first, shortly after 40 °C, followed by the duplex with A21_1MUT at ∼55 °C (Fig. 6C). The duplex with the fully complementary sequence showed high stability in the studied temperature range with only the onset of melting at the highest studied temperature (70 °C). In the case of the 100% coding DNA-NPs, the duplex with A21_3MUT started melting at 50 °C, whereas the A21_1MUT duplex started melting after 60 °C (Fig. 6C). The A21 duplex was stable in the studied temperature range, showing no noticeable changes. Undoubtedly, introducing point mutations drastically decreased the melting temperature for our DNA-NPs, which showed their good sensitivity to mutations.

According to the above-mentioned results, we can see that both 10% and 100% coding DNA-NPs have the capacity to spot mutations, but to different extents. In the case of the 10% coding DNA-NPs, the melting temperatures were systematically lower than the 100% coding ones, allowing more sensitive detection in the normal operational temperature range of the DNA-NPs. Indeed, at 60 °C, 10% DNA-NPs could clearly distinguish three mutations, whereas at 70 °C, even a single-point mutation was clearly spotted. In the case of the 100% coding DNA-NPs, in the working temperature range it is still possible to distinguish the sample with three mutations, but for one mutation it cannot be done because of its too high melting point.

We came to the conclusion that despite the strong DNA cooperativity effects, our DNA-NPs are capable of distinguishing mutations, even a single one in a 21 nt-long oligonucleotide. Given that a certain temperature range has to be maintained because of the nature of the nanoprobes, the 10% coding DNA-NPs are a good fit to be used for the detection of sequences with mutations. The presence of DNA-NPs improved the sensitivity to mutations at higher temperatures, but the exact temperature that has to be applied to distinguish one or more mutations has to be defined for each target, given that the *T*_m_ depends on the sequence.

### Kinetics of hybridization and intrinsic sensitivity of the system

Thus far, we were focused on the stability of the obtained duplexes of DNA-NPs, which is essentially a thermodynamic parameter. Conversely, the kinetics of the process is also very important, especially in the application of DNA/RNA sensing, where the target concentration can vary. Therefore, we investigated how fast the DNA-NPs capture their target sequence at room temperature (∼22 °C) and what is the lowest concentration of target DNA that can be robustly detected.

In this study, DNA-NPs were mixed with the DNA-acceptor at different concentrations and the kinetics of the hybridization was monitored as a change in the FRET signal. Based on the above-mentioned results, 100% coding DNA-NPs and 21-nt DNA-acceptor (A21) were selected to achieve the most efficient hybridization and the maximum duplex stability. The ratio of DNA-NPs : A21 was kept at 1 : 10, while the concentration of the components was decreased gradually to 1 pM of DNA-NPs and 10 pM of A21. After both components were mixed, the fluorescence spectra of each mixture were recorded immediately, and then at 1 min intervals up to 10 min.

**Fig. 7:**
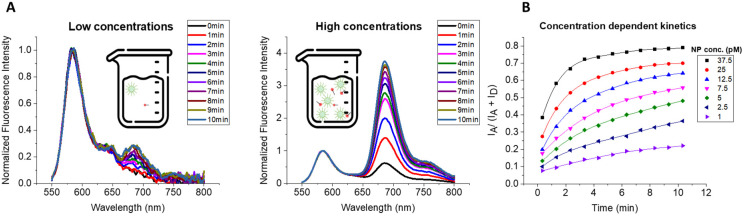
Kinetics of hybridization. (A) Normalized (and smoothed) fluorescence spectra for 100% coding DNA-NPs mixed with the acceptor-sequence with 21 nucleotides at a ratio of 1 NP : 10 21-nt acceptor in low (1 pM DNA-NPs) and high (37.5 pM DNA-NPs) concentrations. The spectra were recorded immediately after mixing for a duration of 10 minutes at the frequency of one spectrum per minute. Schematic representation of the DNA-NPs and acceptor-sequences in solution at different concentrations. (B) FRET ratios for varying concentrations of 100% coding DNA-NPs: 21-nt acceptor up to 10 minutes. The curves connecting the data points are the polynomial fits. In this experiment, the ratio of 1 NP :10 21-nt acceptor was maintained throughout, starting with 1 pM of DNA-NPs hybridized with 10 pM of the 21-nt acceptor and increasing the concentration. Mg buffer was systematically used. Spectra were measured at 530 nm excitation.

Due to the fast acquisition time required to capture each time point, the spectra were quite noisy for the lowest concentration (Fig. 7A). Therefore, the spectra were smoothed to properly calculate the FRET ratio, which was then plotted against time for each sample (Fig. 7B). In all the studied mixtures, the FRET ratio increased over time, showing that the DNA-acceptor hybridized on the DNA-NP surface, leading to an increase in the FRET signal (Fig. 7B). The kinetics of the FRET response (*i.e.* DNA hybridization) showed a clear concentration dependence. The increase in the FRET signal was the steepest for the most concentrated sample (37.5 pM of DNA-NPs), with the reaction completing within 5 min, and the slowest for the most diluted ones (at 1 pM of DNA-NPs). It was observed that the initial speed of hybridization was higher and it decreased over time, which was especially well seen for higher concentrations. Therefore, the kinetics curve could be fitted with a polynomial function (Table S2). This is typical for bimolecular processes where both components are consumed over time, and thus the reaction rate decays non-linearly. Several conclusions can be drawn from these measurements. Firstly, we achieved the direct detection of DNA hybridization at a concentration down to 10 pM of the DNA target, which is much lower than that possible for typical fluorescence-based molecular hybridization assays (typically in nM range). This became possible due to the fluorescence signal amplification through the light-harvesting from our DNA-NPs, where the hybridization of a single acceptor leads to >100-fold amplification because of the efficient FRET from hundreds of encapsulated dyes to a single acceptor.^26,27^ Measurements at these low concentrations revealed a key feature of the direct hybridization assay, *i.e.*, the hybridization between DNA-NPs and the complementary sequence (target) is fast (time scale of minutes) and efficient at concentrations of the target and nanoprobe of ≥100 and ≥10 pM, respectively. Below these concentrations, the reaction becomes slow, being controlled by the diffusion of the two components. This defines the limit of detection (LoD) of a direct hybridization assay when the kinetics is diffusion controlled. To measure the LoD of our system, we performed titration of DNA-NPs (100% encoding survivin capture) with the corresponding survivin-encoding DNA fragment bearing acceptor (target). The FRET signal gradually increased with the concentration of the target DNA (Fig. S19A). Based on the calibration curve of FRET ratio *vs.* target DNA concentration (Fig. S19B and C) and the criteria of three standard deviations of the negative control of DNA-NPs without the target DNA, the obtained LoD value was 0.3 pM.

These results will be useful when setting the parameters for final DNA/RNA sensing assays given that they give important insight into the lowest concentration of DNA-NPs that needs to be used and the minimum time required to complete the hybridization. The obtained results shed light on our previous results, where our FRET assays based on similar DNA-NPs at 10 pM concentration achieved an LoD of 1–5 pM for an incubation time in the range of 1–20 h (Table S3).^26,55,58^ In fact, our system exhibits an even better LoD value within a shorter assay time (Table S3). This could be explained by multiple factors, as follows: (1) the use of 100% coding DNA-NPs *vs.* only 1–2% coding DNA-NPs in the above-mentioned studies; (2) FRET turn-on response in the presence of the target *vs.* FRET turn-off in the above-mentioned studies; and (3) slightly higher temperature used of 40 °C *vs.* 4–30 °C. Nevertheless, similar to the previous studies, the sensitivity remains limited to the pM regime. This shows that when a further improvement in sensitivity is needed, the concentration of one of the two hybridization components should be higher than the above-mentioned limits. For example, fM sensitivity to the DNA/RNA targets was recently achieved in our direct hybridization assay, where DNA-NPs were used at a concentration of 200 pM.^61^

## Conclusions

DNA-functionalized polymeric NPs have emerged as powerful nanomaterials within the class of SNA, which enable the ultrasensitive detection and imaging of nucleic acids in solutions and cells owing to their high fluorescence brightness. Herein, we addressed a fundamental unexplored question on polymeric DNA-NPs: how do oligonucleotides grafted on the surface of polymeric NPs at a high density impact their capacity to specifically hybridize with complementary sequences and how stable are the obtained duplexes? To address this, we employed FRET between DNA-NPs as donors and labelled complementary strands of varying length as the acceptor. It was found that the DNA on the NP surface exhibits a dramatic enhancement in duplex stability compared to the free DNA duplexes (>20 °C), which is also accompanied by an enhanced sharpness in the melting curve of the former. These effects increase with an increase in the density of DNA on the NP surface, which suggests that a DNA cooperativity effect is responsible for the enhancement in duplex stability and sharpness of the melting curves. As a result, the DNA-NPs were able to capture even short DNA duplexes down to 8 nt at RT, which are unstable under these conditions. The observed cooperativity effect has been previously shown for SNA based on gold NPs.^29,70^ Indeed, in these reports, the melting curves for the duplexes with SNA were sharper compared to the individual DNA strands, although the enhancement in the melting temperature was lower than that in the present study.^29,70^ The phenomenon of DNA cooperativity stems from at least two factors. First one is the high local concentration of DNA grafted at high density on the NP surface. This is in line with the fact that the measured melting temperature is lower for 10% coding DNA-NPs *vs.* 100% coding DNA-NPs. Secondly, the DNA strands are aligned closely on the NP surface, which ensures favorable conditions for a dehybridized complementary stand to re-hybridize with its neighbor capture stand. Furthermore, it was shown that DNA-NPs are capable of distinguishing mutations, even at the single-nucleotide level within the 21 nt sequence, when the appropriate hybridization temperature is used. Given that DNA cooperativity makes the melting curves sharper compared to individual DNA strands,^29,70^ at a temperature close to their melting point, DNA-NPs should be even more sensitive to DNA mutations than the individual DNA strands. The hybridization between DNA-NPs and the complementary sequences is fast (min time scale) at probe and target concentrations of ≥10 and ≥100 pM, respectively. Below that, the reaction takes much longer time, which indicates the importance of the use of a sufficiently concentrated nanoprobe in the development of fast and sensitive assays for DNA/RNA target detection. Nevertheless, the achieved limit of detection (LoD) in this model system reached 0.3 pM. These observations explain previous results on FRET-based nanosensors, where the observed limited sensitivity in the low-pM range is linked to the slow kinetics of DNA hybridization at too low concentrations of DNA nanoprobe and nucleic acid target.^26,55,58^ The current model system presents some advantages in terms of LoD and detection time (20 min) because it uses 100% coding sequences on NPs with higher incubation temperature (40 °C) and operates based on the turn-on FRET mechanism. Overall, this work shows that polymeric DNA-NPs present enhanced capacity to hybridize with complementary stands compared to individual DNA, and at the same time well distinguish a single mutation at the appropriate temperature. Moreover, it provides insights into the design of DNA-NPs to obtain better nucleic acid sensing assays.

## Conflicts of interest

ASK is the co-founder of BrightSens Diagnostics SAS and PG was employed by this company. ASK and PG submitted patent applications related to the described technology.

## Supplementary Material

NR-017-D5NR01614B-s001

## Data Availability

The data supporting this article have been included as part of the SI. Additional data on characterization of DNA-NPs by DLS, TEM and fluorescence spectroscopy, details on data fitting, titration results and comparison of the assay performance. See DOI: https://doi.org/10.1039/d5nr01614b.
