# Supplementary material for: Cooperative effects in DNA-functionalized polymeric nanoparticles
Source: Nanoscale. 2025 Aug 13;17(36):21083–95. doi: 10.1039/d5nr01614b (PMC12394930; doi:10.1039/d5nr01614b)
Supplement: NR-017-D5NR01614B-s001 [file NR-017-D5NR01614B-s001.pdf]

Supplementary Information (SI) for Nanoscale.
This journal is © The Royal Society of Chemistry 2025

Supplementary information

Cooperative effects in DNA-functionalized polymeric nanoparticles

Paraskevi Gaki,[1,2] and Andrey S. Klymchenko[1,*]

[1] Laboratoire de Bioimagerie et Pathologies, UMR 7021 CNRS, Faculté de Pharmacie, Université de Strasbourg, 67401 Illkirch, France

[2] BrightSens Diagnostics SAS, 11 Rue de l'Académie, 67000 Strasbourg, France

*Corresponding author, email: andrey.klymchenko@unistra.fr

Table S1. Hydrodynamic diameter and polydispersity (PdI) by DLS and the diameter by TEM of fluorescent NPs used in this work.

**NPs name**	**Size by DLS (nm)**	**PdI**	**Size by TEM (nm)**
Bare NPs	33 ± 2	0.13 ± 0.01	23 ± 4
100% coding DNA-NPs	46 ± 1	0.11 ± 0.01	28 ± 5
10% coding DNA-NPs	45 ± 2	0.12 ± 0.01	32 ± 6

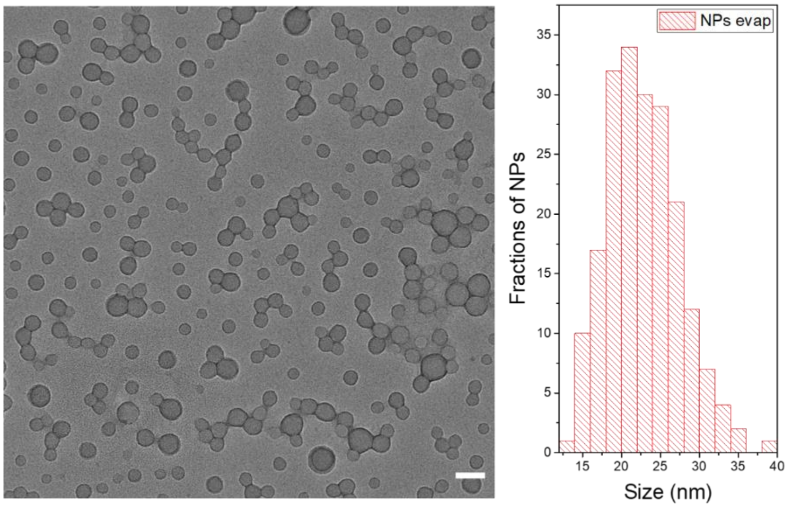

Figure S1. TEM image (left) and size distribution diagram (right) of bare PEMA-AspN3 NPs loaded with 33 wt% of R18/F5-TPB dye (with respect to total NP mass). Scale bar: 50 µm.

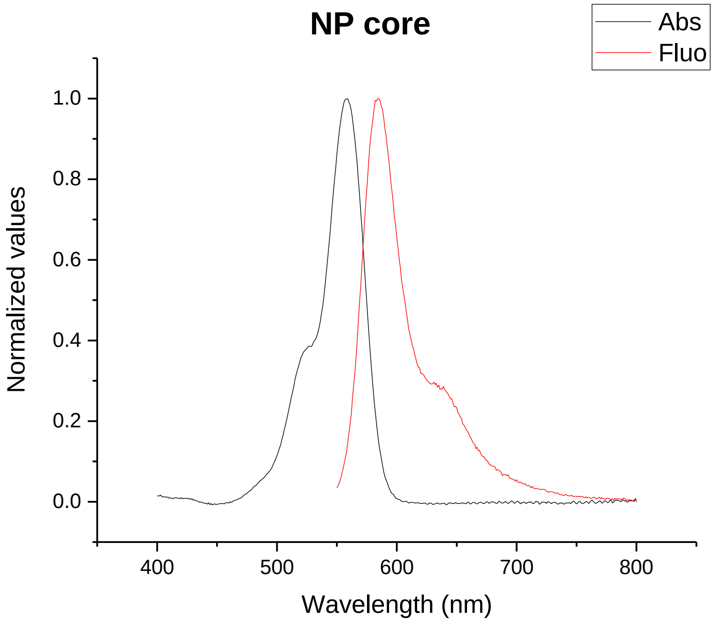

Figure S2. Absorption and emission spectra of bare polymeric (PEMA-AspN3) NPs loaded with R18/F5-TPB at 33 wt% (vs total NP mass).

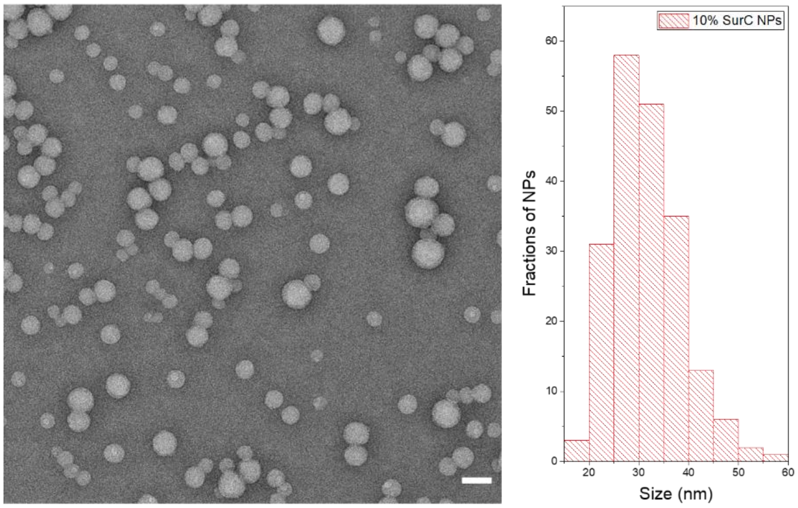

Figure S3. TEM image (left) and size distribution diagram (right) PEMA-AspN3 NPs functionalized at 10% with survivin capture coding sequence and 90% A20 (non-coding) sequence and loaded with 33 wt% of R18/F5-TPB dye (with respect to total NP mass). Scale bar: 50 µm.

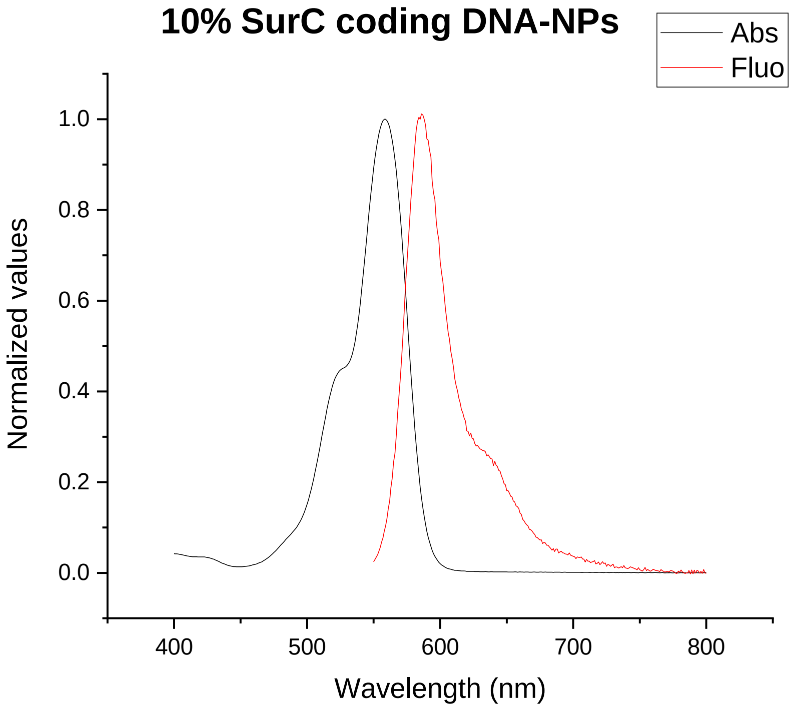

Figure S4. Absorption and emission spectra of dye-loaded polymeric DNA-NPs coated with 10% SurC and 90% A20 oligonucleotides (10% coding DNA-NPs).

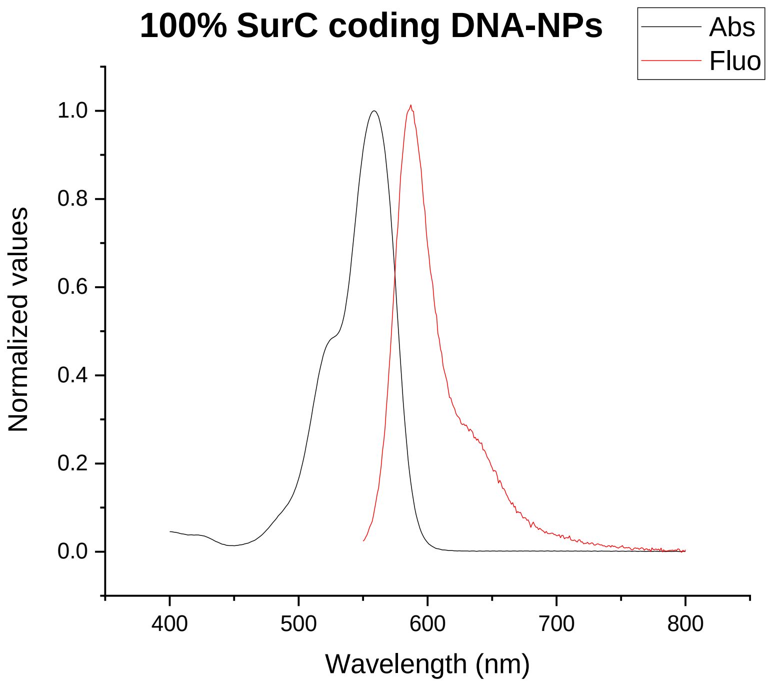

Figure S5. Absorption and emission spectra of dye-loaded polymeric DNA-NPs coated with SurC oligonucleotide (100% coding DNA-NPs).

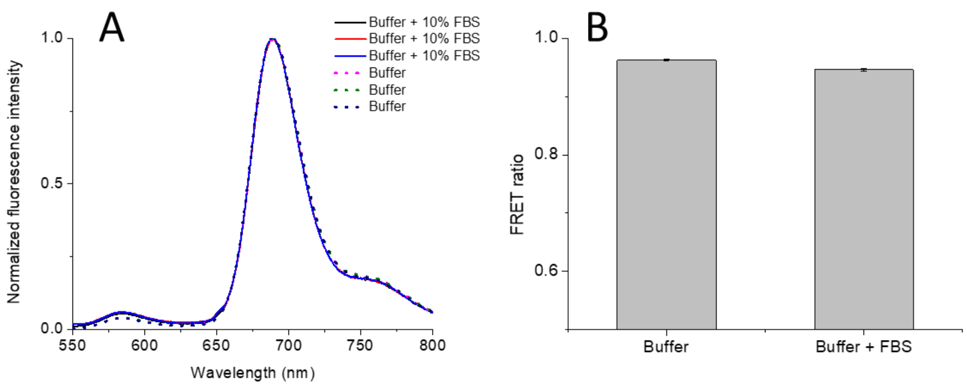

Figure S6. Effect of fetal bovine serum (FBS) on FRET response of DNA-NPs to complementary DNA strand. Fluorescence spectra (A) and FRET ratio values of 100% coding (100% survivin capture sequence) DNA-NPs (50% wt% of R18/F5-TPB dye with respect to the polymer, meaning 33 wt% of the total mass of the NPs), hybridized at 40°C for 20 min with an acceptor dye bearing DNA survivin fragment sequence of 21 nucleotides, fully complementary to the capture sequence on the DNA-NPs. 100 pM of DNA-NPs were hybridized with 10 nM of the 21-nt acceptor-sequence. Spectra were measured at 530 nm excitation at RT. Error bars correspond to standard deviation of the mean (n = 3).

THERMAL STABILITY

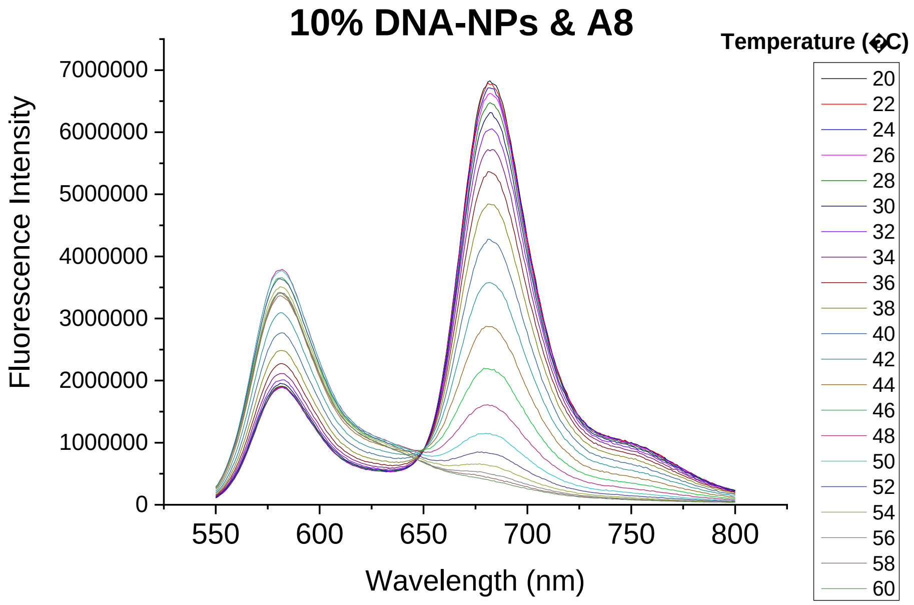

Figure S7. Fluorescence spectra of 10% coding DNA-NPs mixed with 8-nt acceptor at different temperatures.

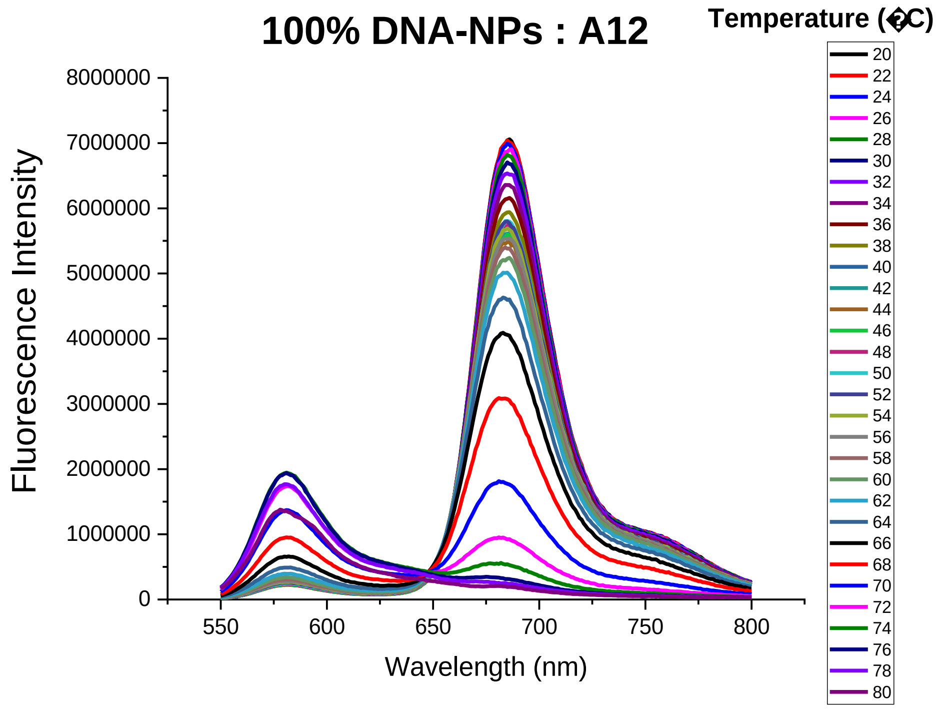

Figure S8. Fluorescence spectra of 100% coding DNA-NPs mixed with 12-nt acceptor at different temperatures.

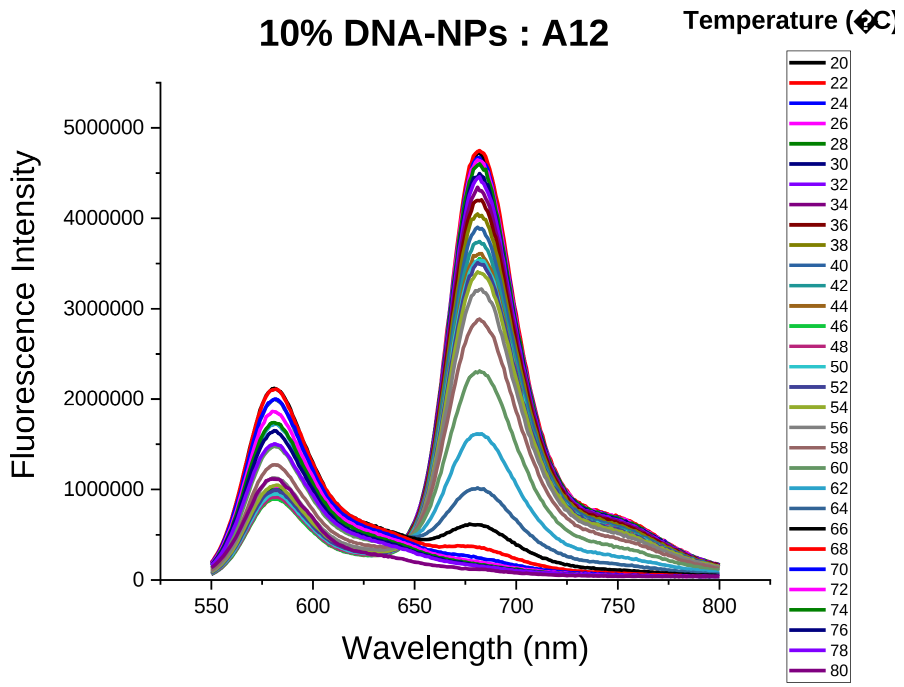

Figure S9. Fluorescence spectra of 10% coding DNA-NPs mixed with 12-nt acceptor at different temperatures.

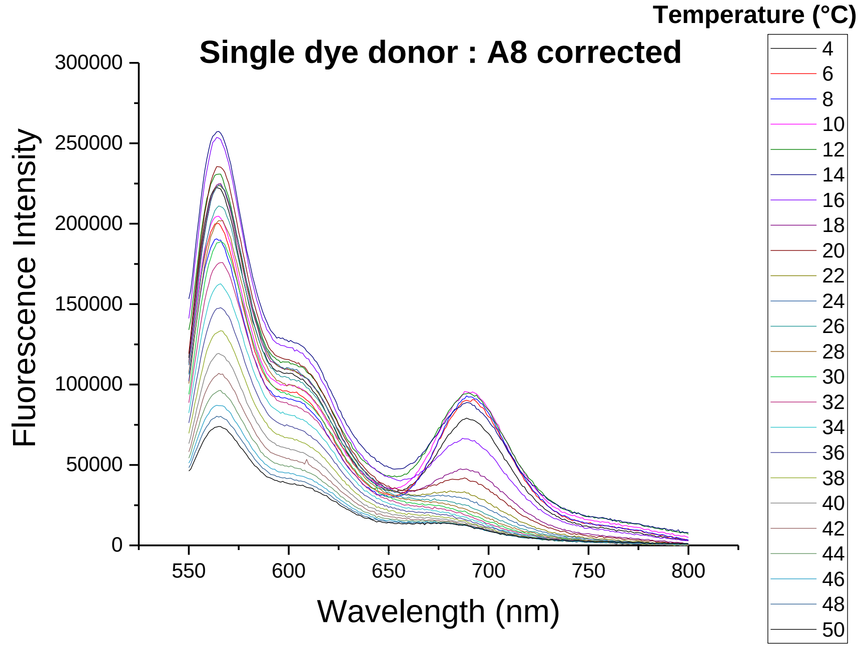

Figure S10. Fluorescence spectra of donor dye-labeled SurC oligonucleotide mixed with 8-nt acceptor at different temperatures.

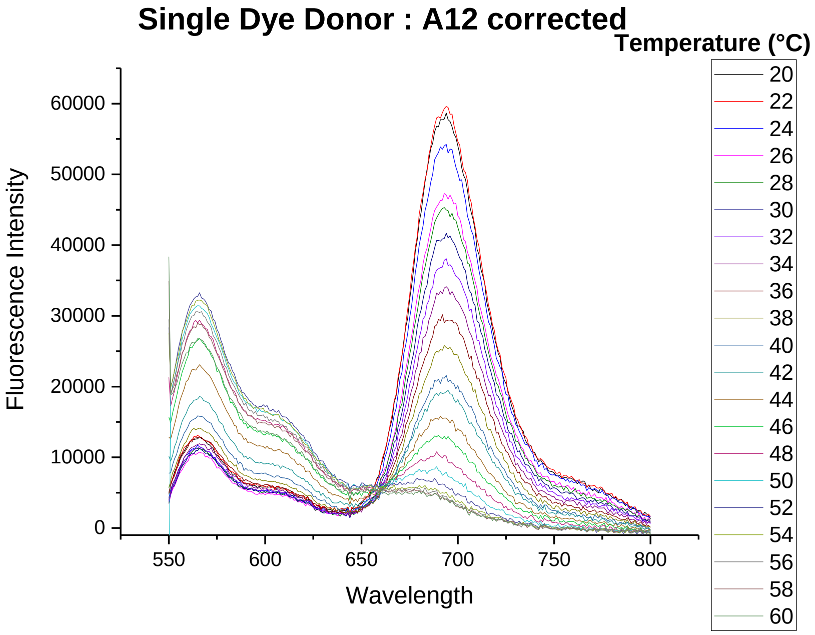

Figure S11. Fluorescence spectra of dye-labeled SurC oligonucleotide mixed with 12-nt acceptor at different temperatures.

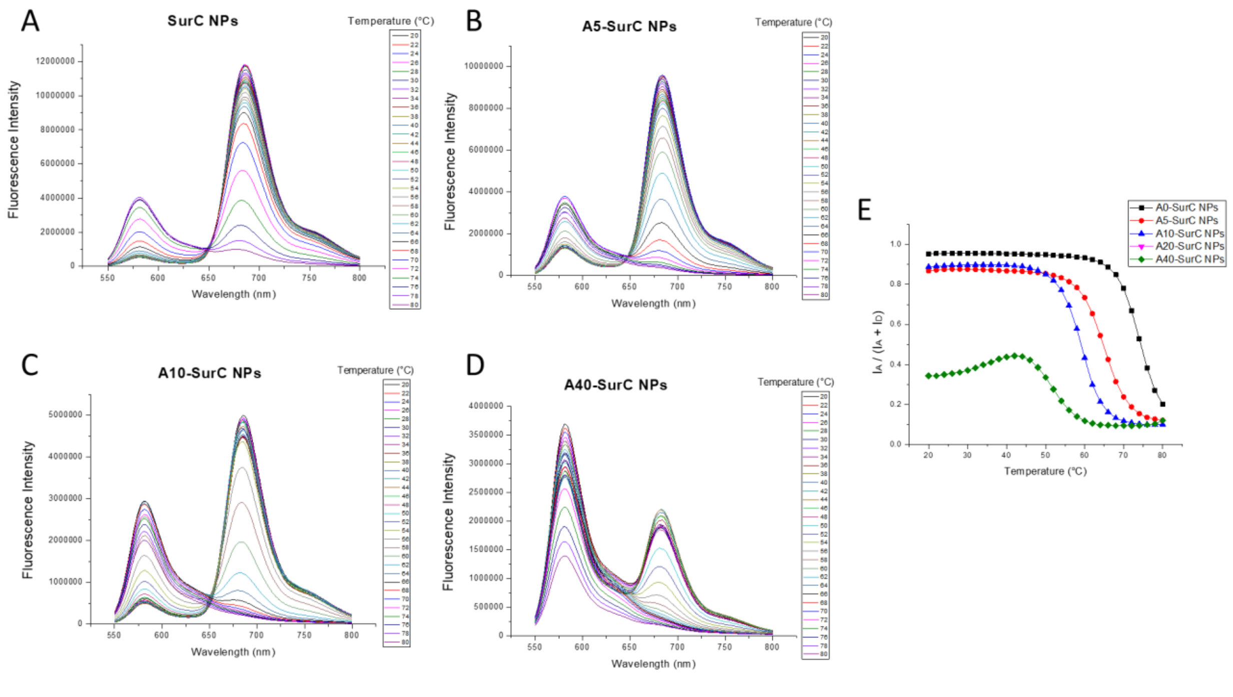

Figure S12. (A-D) Fluorescence spectra of 100% coding DNA-NPs mixed with 12-nt acceptor at different temperatures. The coding sequence was grafted to the NPs surface at different distances: 0 nt (SurC NPs), 5 nt (A5-SurC), 10 nt (A5-SurC) and 40 nt (A5-SurC). (E) Corresponding FRET ratio values vs temperature.

MUTATIONS

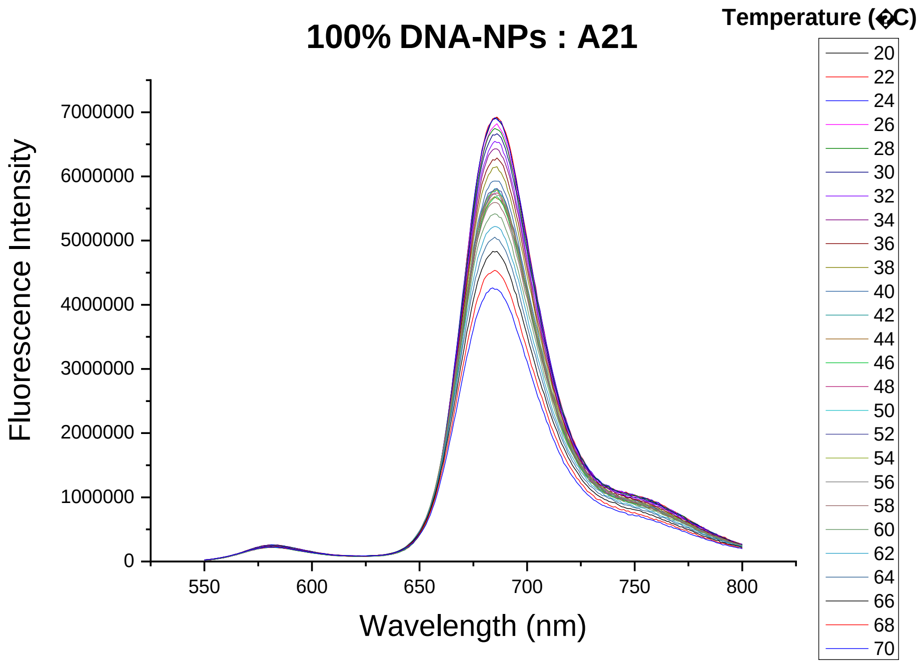

Figure S13. Study of mutations with 100% DNA-NPs mixed with 21-nt acceptor: fluorescence spectra at different temperatures.

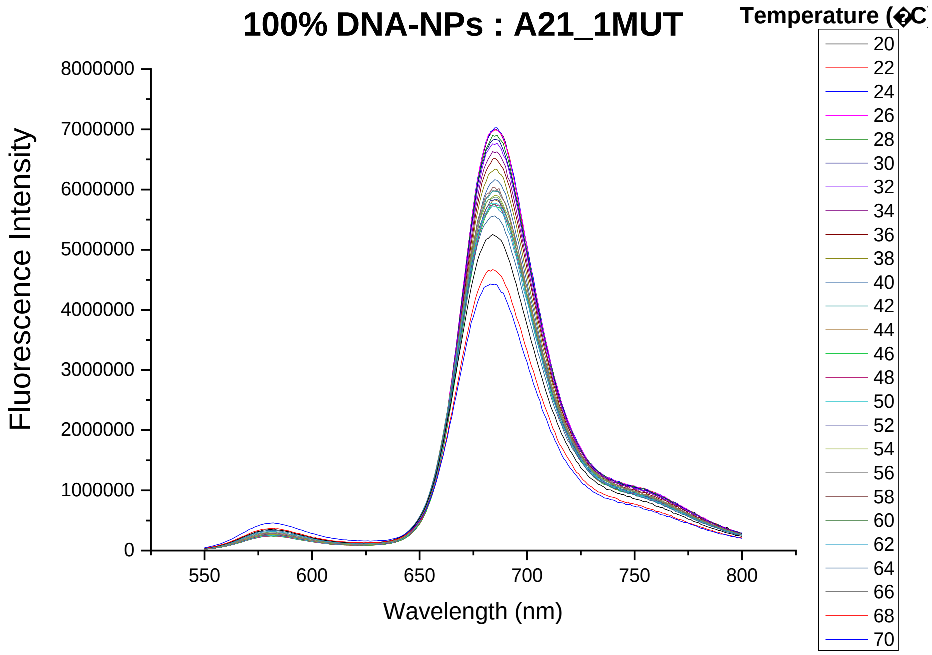

Figure S14. Study of mutations with 100% DNA-NPs mixed with 21-nt acceptor with 1 mutation: fluorescence spectra at different temperatures.

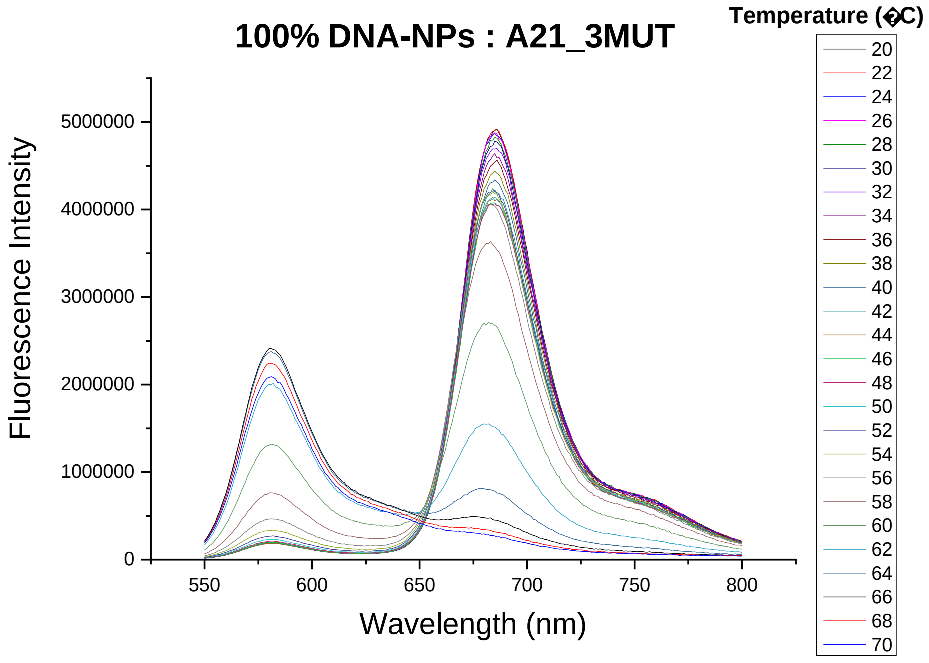

Figure S15. Study of mutations with 100% DNA-NPs mixed with 21-nt acceptor with 3 mutations: fluorescence spectra at different temperatures.

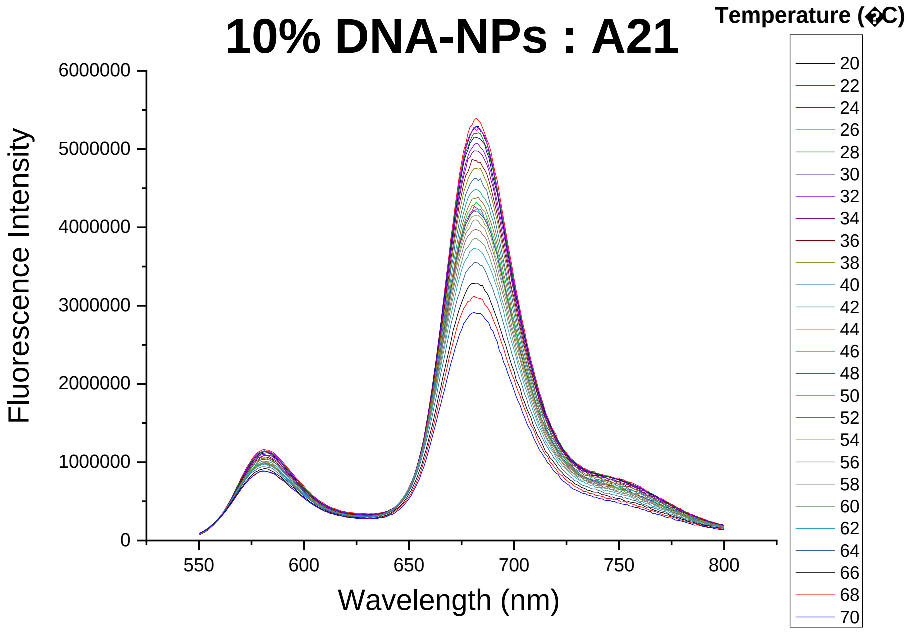

Figure S16. Study of mutations with 10% DNA-NPs mixed with 21-nt acceptor: fluorescence spectra at different temperatures.

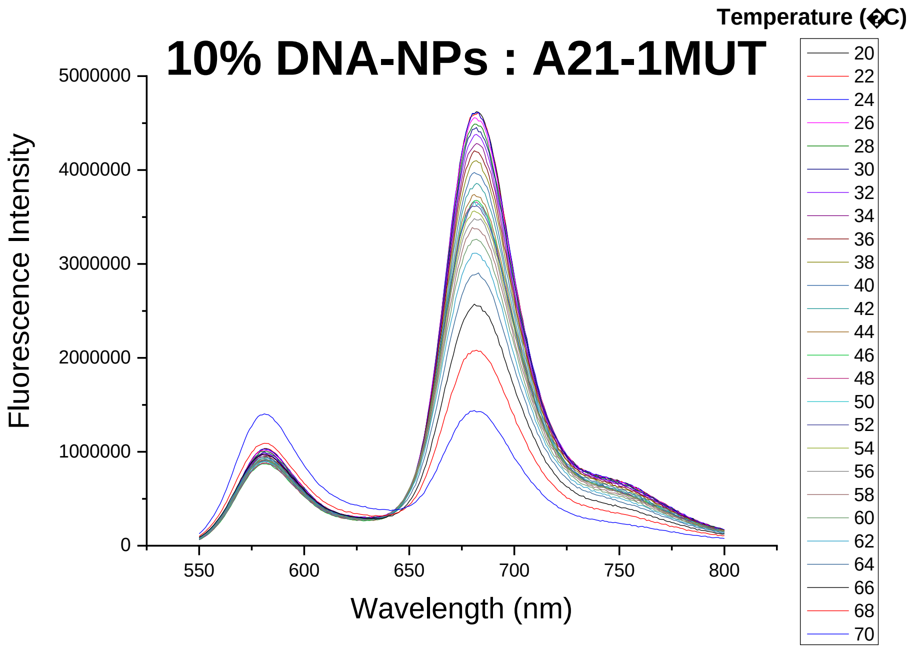

Figure S17. Study of mutations with 10% DNA-NPs mixed with 21-nt acceptor with 1 mutation: fluorescence spectra at different temperatures.

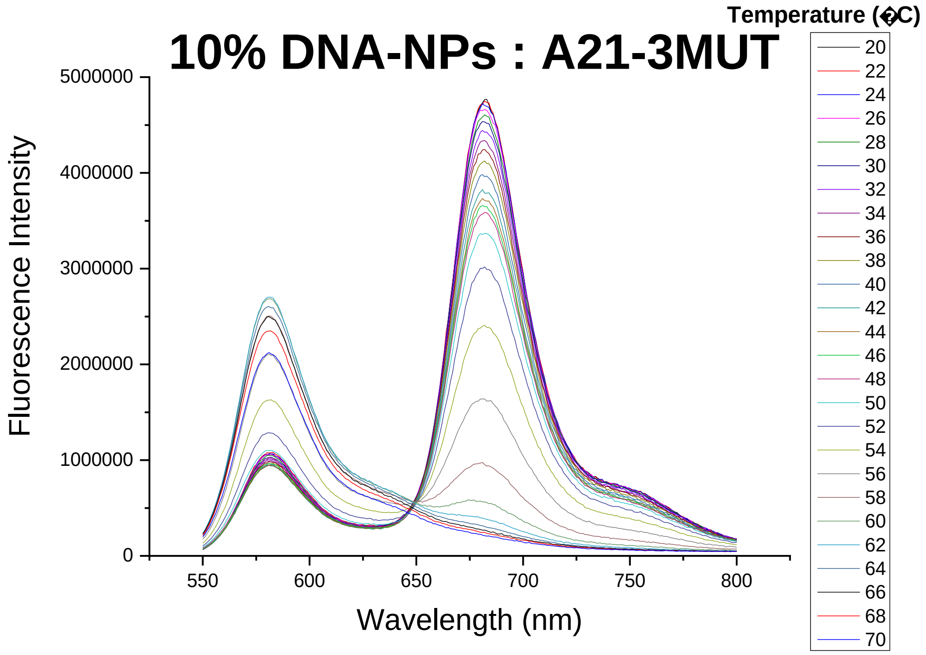

Figure S18. Study of mutations with 10% DNA-NPs mixed with 21-nt acceptor with 3 mutations: fluorescence spectra at different temperatures.

KINETICS analysis

Table S2. Parameters of the fit for the kinetics curves in Figure 7.

Equation	y = Intercept + B1*x^1 + B2*x^2 + B3*x^3 + B4*x^4 + B5*x^5		
Weight	No Weighting		
Residual Sum of Squares	1.05897E-4		
Adj. R-Square	0.9912		
		Value	Standard Error
1	Intercept	0.06656	0.00804
	B1	0.02618	0.0166
	B2	-0.0027	0.0099
	B3	5.46212E-4	0.00238
	B4	-6.53053E-5	2.46534E-4
	B5	2.65807E-6	9.2116E-6

Equation	y = Intercept + B1*x^1 + B2*x^2 + B3*x^3 + B4*x^4 + B5*x^5		
Weight	No Weighting		
Residual Sum of Squares	1.72504E-4		
Adj. R-Square	0.99496		
		Value	Standard Error
2.5	Intercept	0.07625	0.01026
	B1	0.0762	0.02119
	B2	-0.01544	0.01263
	B3	0.00205	0.00303
	B4	-1.26987E-4	3.14655E-4
	B5	2.84518E-6	1.17569E-5

Equation	y = Intercept + B1*x^1 + B2*x^2 + B3*x^3 + B4*x^4 + B5*x^5		
Weight	No Weighting		
Residual Sum of Squares	7.40255E-5		
Adj. R-Square	0.99881		
		Value	Standard Error
5	Intercept	0.10381	0.00672
	B1	0.08856	0.01388
	B2	-0.01006	0.00828
	B3	5.28587E-4	0.00199
	B4	9.69335E-6	2.06123E-4
	B5	-1.34143E-6	7.70165E-6

Equation	y = Intercept + B1*x^1 + B2*x^2 + B3*x^3 + B 4*x^4 + B5*x^5		
Weight	No Weighting		
Residual Sum of Squares	1.18032E-4		
Adj. R-Square	0.99847		
		Value	Standard Error
7.5	Intercept	0.14612	0.00848
	B1	0.09742	0.01753
	B2	-0.0099	0.01045
	B3	5.86435E-4	0.00251
	B4	-2.1711E-5	2.60277E-4
	B5	5.27356E-7	9.72508E-6

Equation	y = Intercept + B1*x^1 + B2*x^2 + B3*x^3 + B4 *x^4 + B5*x^5		
Weight	No Weighting		
Residual Sum of Squares	5.24884E-5		
Adj. R-Square	0.99946		
		Value	Standard Error
12.5	Intercept	0.13795	0.00566
	B1	0.1965	0.01169
	B2	-0.04316	0.00697
	B3	0.00577	0.00167
	B4	-4.05088E-4	1.73567E-4
	B5	1.13032E-5	6.48521E-6

Equation	y = Intercept + B1*x^1 + B2*x^2 + B3*x^3 + B4 *x^4 + B5*x^5		
Weight	No Weighting		
Residual Sum of Squares	1.49949E-5		
Adj. R-Square	0.99983		
		Value	Standard Error
25	Intercept	0.20204	0.00302
	B1	0.24069	0.00625
	B2	-0.05916	0.00372
	B3	0.00812	8.93953E-4
	B4	-5.68507E-4	9.27697E-5
	B5	1.56658E-5	3.46628E-6

Equation	y = Intercept + B1*x^1 + B2*x^2 + B3*x^3 + B 4*x^4 + B5*x^5		
Weight	No Weighting		
Residual Sum of Squares	9.36831E-5		
Adj. R-Square	0.99876		
		Value	Standard Error
37.5	Intercept	0.28748	0.00756
	B1	0.32466	0.01562
	B2	-0.0977	0.00931
	B3	0.01517	0.00223
	B4	-0.00116	2.31881E-4
	B5	3.44061E-5	8.6641E-6

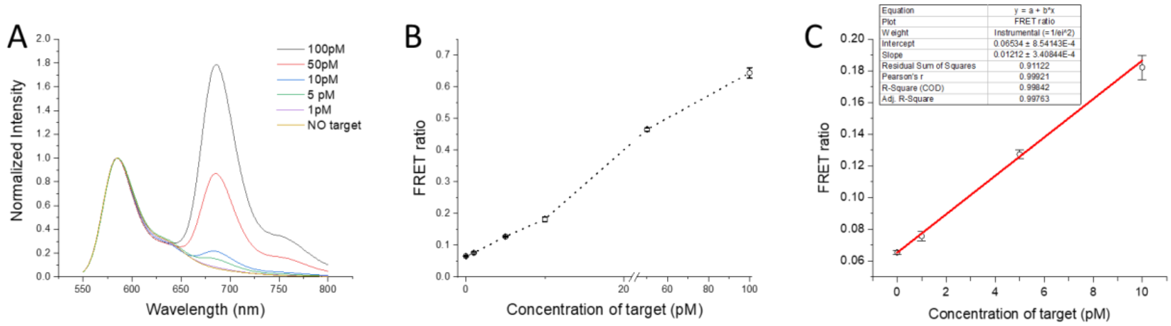

Figure S19. (A) Fluorescence spectra of 100% coding (survivin capture sequence) DNA-NPs (50% wt% of R18/F5-TPB dye with respect to the polymer, meaning 33 wt% of the total mass of the NPs), with increasing concentrations of an acceptor dye bearing DNA survivin fragment sequence of 21 nucleotides (hybridized at 40°C for 20 min with). 100 pM of DNA-NPs were used. Spectra were measured at 530 nm excitation at RT. (B) Corresponding titration curve of FRET ratio vs target concentration used for calculation of the limit of detection. Error bars correspond to standard deviation of the mean (n = 3). (C) Linear fit for the concentration range 0-10 µM of the oligonucleotide target, used for calculation of the limit of detection. The following linear calibration function was obtained: FRET ratio = 0.653 + 0.0121 × [C], where [C] is concentration of the oligonucleotide target in pM.

Table S3. Comparison of the performance of the FRET assay of the present study with previously reported assays based on similar DNA-NPs.

Assay	Time of assay and temperature	Limit of detection
40 nm DNA-NPs with survivin fragment	20h at 4 °C	5 pM
20 nm DNA-NPs with survivin with survivin fragment	3h at 20 °C	2 pM
20 nm DNA-NPs for microRNA	6h at 30 °C	1.3-4.4 pM
20 nm DNA-NPs (present study)	20 min at 40 °C	0.3 pM
